# Chemical Composition and Bioactivities of Turkish *Leonurus* Species (Lamiaceae) Extracts: Antioxidant, Antimicrobial, and Antiproliferative Potential

**DOI:** 10.3390/molecules31101708

**Published:** 2026-05-18

**Authors:** Nagehan Saltan, Yavuz Bülent Köse, Fatih Göger, Derya Osmaniye, Gökalp İşcan

**Affiliations:** 1 Department of Pharmaceutical Botany, Faculty of Pharmacy, Anadolu University, Eskişehir 26470, Türkiye; ybkose@anadolu.edu.tr; 2 Department of Pharmaceutical Botany, Faculty of Pharmacy, Afyonkarahisar Health Sciences University, Afyonkarahisar 03030, Türkiye; fatih.goger@afsu.edu.tr; 3 Department of Pharmaceutical Chemistry, Faculty of Pharmacy, Anadolu University, Eskişehir 26470, Türkiye; dosmaniye@anadolu.edu.tr; 4 Department of Pharmacognosy, Faculty of Pharmacy, Anadolu University, Eskişehir 26470, Türkiye; giscan@anadolu.edu.tr

**Keywords:** *Leonurus*, phytochemical profiling, HPLC-MS/MS, antioxidant activity, antiproliferative activity

## Abstract

The genus *Leonurus* L. is renowned for its diverse secondary metabolites with significant pharmacological value; however, the chemical biodiversity and biological potential of its indigenous members in Türkiye remain largely unexplored. This study investigates four species (*L. cardiaca*, *L. quinquelobatus*, *L. glaucescens*, and *L. persicus*) to elucidate their phytochemical architecture and therapeutic capacities. Characterization of the ethanol, methanol, and aqueous (5% infusion) extracts via HPLC-MS/MS identified verbascoside, genkwanin, and caffeoylquinic acids as the major representative bioactive constituents across the studied *Leonurus* species. The extracts exhibited measurable biological activity, with *L. cardiaca* displaying the highest antioxidant profile (EC_50_ 0.117 ± 0.01 mg/mL for DPPH, 2.731 ± 0.01 mM/Trolox for ABTS), correlating with its phenolic content. Notably, the extracts demonstrated notable anticandidal activity (MIC 0.1–1 mg/mL) and negligible to moderate antibacterial effects, alongside varying levels of susceptibility against breast (MCF7) and glioma (C6) cancer cell lines. These effects showed differentiation in toxicity compared to lung (A549) cells. This investigation provides scientific evidence supporting the traditional medicinal use of *Leonurus* species while highlighting their potential as standardized sources for the pharmaceutical and nutraceutical sectors. Our results lay a robust foundation for future bioactivity-guided isolation studies to further elucidate the molecular mechanisms behind their differential biological effects.

## 1. Introduction

The *Lamiaceae* Martinov family is highly significant due to its aromatic, medicinal, and essential oil-producing species. While this family is distributed across the Mediterranean and subtropical regions worldwide, its members are predominantly found in southern Türkiye, especially in the hilly areas of the Mediterranean. The genus *Leonurus* L. has been recognized since ancient times for its medicinal properties; it acts as a regulator of the central nervous and cardiovascular systems, providing a sedative effect that helps manage heart and lung conditions [1]. The genus *Leonurus* L. is represented by 24–25 species worldwide according to the World Flora Online database; while native to Eurasia, it has become naturalized across the Americas, Africa, and the Pacific. Among these, *L. cardiaca*, *L. japonicus*, and *L. sibiricus* are the most prominent species used in traditional and modern medicine globally [2]. According to the Flora of Turkey [3], four species of the *Leonurus* genus are found in Türkiye: *L. cardiaca* L., *L. glaucescens* Bunge, *L. persicus* Boiss., and *L. quinquelobatus* Gilib. These species are geographically distributed across the Marmara, Thrace, and Eastern Anatolia regions, where they occur as part of the natural vegetation [4]. *L. cardiaca*, commonly known as “Motherwort, Lion’s Tail, lion grass, lion’s tail, aslankuyruğu,” has been extensively documented in the literature [4,5]. The European Medicines Agency (EMA) recommends both aqueous and ethanol extracts of *L. cardiaca* as medicinal preparations, which can be selectively enriched using different extraction techniques [6]. The species, traditionally used to treat nervous and functional cardiac disorders, is officially recognized in pharmacopeias for its sedative, hypotensive, and cardiotonic pharmacological effects [6,7,8]. *L. glaucescens* is a perennial plant with a purplish, densely hairy stem characterized by backward-curved hairs at its corners [3]. An ethnobotanical study conducted in Eastern Anatolia reported that the root of *L. glaucescens*, locally known as “Öküzguyruğu,” is used as a poultice for inflamed wounds and as an infusion for heart diseases [9]. *L. quinquelobatus* (five-lobed Motherwort) is used for increasing cardiac activity, for cardiac neurosis, gastrointestinal and nervous diseases, headaches, and cough, as well as for the treatment of rheumatism, weakness, asthma, and as a diuretic and menstrual cycle regulation tool [10]. Pharmacological studies have linked *Leonurus* species to various medicinal properties. Beyond their antioxidant potential, these species exhibit various pharmacological activities, including anti-inflammatory, anticancer, and antibacterial effects [11,12,13,14,15,16]. In particular, compounds such as quercetin, caffeic acid, lavandulifolioside, verbascoside, chlorogenic acid, rutin, and ursolic acid in *L. cardiaca* extracts contribute to its bioactivity. Angeloni et al. (2021) reported that *L. cardiaca* primarily contains phenolic acids (chlorogenic and caffeic acids), flavonoids (rutin and quercetin), phenylethanoid glycosides (verbascoside and lavandulifolioside), guanidine pseudoalkaloids (leonurin), as well as diterpenes and triterpenes [11].

The pharmaceutical potential of plants is determined by the wide range of biocompounds they synthesize as an adaptive approach to survive environmental stress. Consequently, *Leonurus* species growing in the specific ecological conditions of Türkiye may exhibit distinct chemical profiles and biological activities compared to those originating from different geographical locations. Despite the global ethnomedicinal use of *Leonurus* species for treating various ailments, existing literature primarily focuses on *L. cardiaca* from other regions, leaving the biological potential of the Turkish flora largely unexplored. Furthermore, scientific data regarding the bioactivities of species other than *L. cardiaca* remains remarkably scarce. Driven by this gap in knowledge and the chemical diversity of the genus, the present study was designed to investigate the biological activities of extracts obtained from the aerial parts of four *Leonurus* species indigenous to Türkiye. Our investigation encompasses a comprehensive evaluation of their free radical scavenging capacities, antimicrobial properties, and cytotoxic effects on various cancer cell lines. Additionally, to establish a link between the observed bioactivities and the phytochemical composition, the major constituents were characterized using High-Performance Liquid Chromatography coupled with Mass Spectrometry (HPLC-MS/MS). The main goal of this research is to describe the qualitative phytochemical profile of *Leonurus* species. This study establishes a basic baseline for the genus and its ensuing pharmacological assessments by mapping the structural diversity of its secondary metabolites. This study provides the first integrated report on the phytochemical and biological profiling of *Leonurus* species distributed in Türkiye. Our results demonstrated that *L. cardiaca* possesses the highest phenolic density and antioxidant capacity among the studied taxa, while all studied species showed differential sensitivities across the tested cell lines. These findings provide scientific evidence supporting the traditional uses of the genus and offer new insights into their potential as natural sources of bioactive compounds for the pharmaceutical industry.

## 2. Results

### 2.1. HPLC–MS/MS Analysis

**Figure 1:**
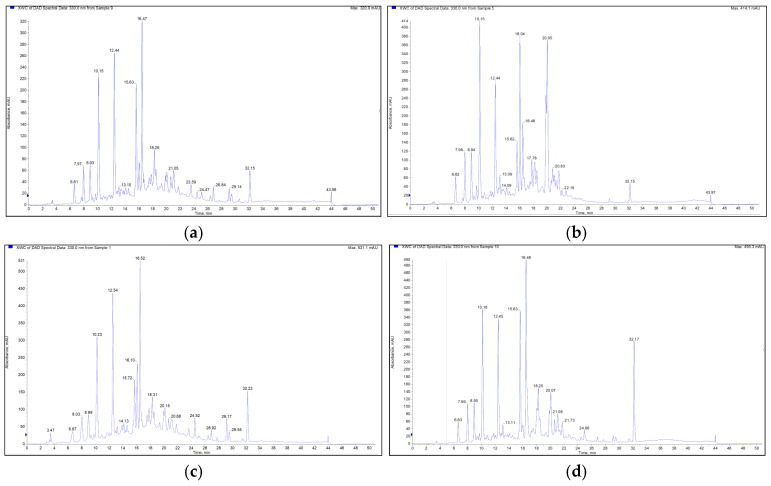
HPLC-DAD (Diode Array Detector) chromatograms (recorded at 330 nm) of *Leonurus* species: (**a**) *L. cardiaca*, (**b**) *L. glaucescens*, (**c**) *L. quinquelobatus*, and (**d**) *L. persicus*. Note: Minor peaks not listed in Table 1 represent unidentified compounds with low-intensity spectral data. The blue square symbol indicates the XWC of DAD spectral data at 330.0 nm.

To qualitatively identify the major constituents in the four Turkish *Leonurus* species, HPLC-MS/MS in negative ionization mode was employed (Table 1). By examining the chromatograms and spectra obtained from the qualitative analysis, the main compounds of *Leonurus* species were identified (Figure 1). As summarized in Table 1, the ethanol, methanol, and aqueous (5% infusion) extracts revealed a rich diversity of secondary metabolites, primarily consisting of phenylethanoid glycosides, hydroxycinnamic acid derivatives, and flavonoids. A total of nine major compounds were identified based on their retention times (Rt), precursor ions ([M-H]^−^), and characteristic MS^2^ fragmentation patterns. In addition to the major peaks identified, several minor peaks were observed at various retention times (e.g., 10.13, 10.19, 18.26, and 24.49 min). These minor phenolics were not tabulated as they exhibited low signal-to-noise ratios or inconsistent MS/MS fragmentation patterns that precluded definitive characterization. Significant variations in chemical biodiversity were observed across the studied species. The presence of verbascoside (*m/z* 622.4) and lavandulifolioside (*m/z* 754.4) was particularly noteworthy, as these phenylethanoid glycosides are chemotaxonomic markers for the Lamiaceae family (Supplementary Figures S1 and S2). Rosmarinic acid (*m/z* 358.7) was uniquely identified in *L. persicus*, suggesting its potential use as a distinguishing marker for this species among the Turkish indigenous members. In contrast, caffeoylquinic acid derivatives (*m/z* 352.7) and quercetin rutinoside (*m/z* 608.4) were ubiquitously present across all four species, regardless of the extraction solvent. To evaluate the extraction efficiency, parallel extractions were performed using ethanol, methanol, and distilled water (5% infusion) independently. This approach explains the presence of major constituents such as verbascoside and caffeoylquinic acids across all extracts, albeit with varying relative abundances. The organic solvent extracts (ethanol and methanol) generally exhibited a broader phytochemical profile compared to infusions, particularly for the flavonoid genkwanin (*m/z* 282.7). Genkwanin, a methylated apigenin derivative, was prominently detected in *L. cardiaca* and *L. quinquelobatus*, which may correlate with the specialized biological activities of these species (Supplementary Figure S3).

### 2.2. Determination of Total Phenolic Contents (TPC), Flavonoid (TFC) Contents

Table 2 displays the findings of the quantitative phytochemical and antioxidant parameters of the investigated *Leonurus* species. The total phenolic contents (TPC), determined via the Folin–Ciocalteu assay and expressed as gallic acid equivalents (GAE), exhibited variability ranging from 41.9 to 170.1 mg GAE/g extract. Notably, the ethanol extracts of *L. cardiaca* (LCE), *L. persicus* (LPE), and *L. glaucescens* (LGE) demonstrated the highest phenolic concentrations, yielding 170.1 ± 0.02, 148.1 ± 0.04, and 143.4 ± 0.03 mg GAE/g, respectively. Regarding flavonoid content, the European Pharmacopoeia establishes a minimum threshold of 0.2% flavonoids for Leonuri cardiacae herba. Our analysis confirmed that the investigated species comply with this pharmaceutical standard (Table 2). The flavonoid content in 1 g of plant material was determined to be 0.26% in *L. quinquelobatus*, 0.35% in *L. glaucescens*, 0.24% in *L. cardiaca*, and 0.31% in *L. persicus*. These results suggest that the Turkish *Leonurus* species align with the flavonoid content requirements defined in international standards.

### 2.3. Antioxidant Capacity: DPPH and ABTS Radical Scavenging Assays

The antioxidant capacity of the plants was assessed using DPPH^•^ and ABTS^•+^ scavenging tests. All extracts displayed dose-dependent antioxidant activity (Table 2). The DPPH scavenging assay revealed that LCE possessed the most potent antioxidant activity with the lowest EC_50_ value of 0.117 mg/mL. In addition, this significant performance was further corroborated by the ABTS assay results, where the highest TEAC (Trolox Equivalent Antioxidant Capacity) was recorded for LCE at 2.73 mM. High antioxidant capacities were also observed for LGE and LPE species and determined as 2.53 mM and 2.25 mM, respectively (Table 2).

**Figure 2:**
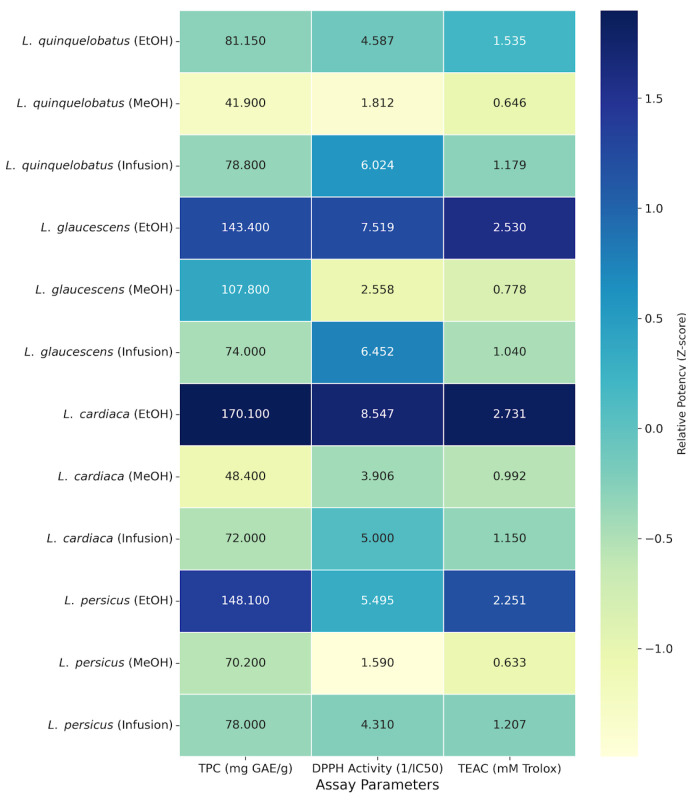
Heatmap analysis of total phenolic content (TPC) and antioxidant capacities (DPPH and TEAC) of four *Leonurus* species. Color intensity reflects standardized Z-scores (relative potency based on 1/EC_50_ values), where darker blue indicates higher phytochemical concentration or stronger antioxidant activity. Numerical values represent raw data (mean ± SD, *n* = 3).

To evaluate the contribution of phenolic constituents to the antioxidant potential, Pearson correlation coefficients were calculated. A strong positive correlation was observed between TPC and TEAC values (r = 0.8873, *p* < 0.001), indicating that phenolic compounds are the primary contributors to the ABTS radical scavenging activity. Furthermore, a moderate negative correlation (r = −0.5007) was found between TPC and DPPH EC_50_ values (Figure 2). This inverse relationship is consistent with the principle that higher phenolic content leads to lower EC_50_, values, suggesting that while phenolics generally enhance DPPH radical scavenging capacity, other secondary metabolites may also influence the specific response in this assay.

### 2.4. Determination of Antimicrobial Activity

The antimicrobial potential of *Leonurus* extracts was evaluated using the broth microdilution method, with results summarized in Table 3 and Table 4. Overall, the investigated extracts exhibited limited antibacterial activity, with MIC values typically ranging from 1000 to ˃8000 µg/mL. Among the samples, the methanolic extract of *L. quinquelobatus* showed relatively lower MIC values against *Pseudomonas aeruginosa*, *Staphylococcus epidermidis*, and *Enterococcus faecalis* compared to other species. The observed efficacy followed the general order of methanol > ethanol > infusion, suggesting that the methanolic solvent may be slightly more effective in extracting constituents with inhibitory potential. Regarding anticandidal activity, the extracts demonstrated moderate inhibitory effects within a concentration range of 0.125 to 2 mg/mL. Notably, the methanol extracts of *L. persicus* exhibited activity against *Candida utilis* and *C. krusei*, both yielding a Minimum Inhibitory Concentration (MIC) of 0.125 mg/mL. These results indicate that while the extracts possess negligible antibacterial effects, they show a more discernible potential against *Candida* species.

### 2.5. In Vitro Cytotoxicity and Antiproliferative Potential

**Figure 3:**
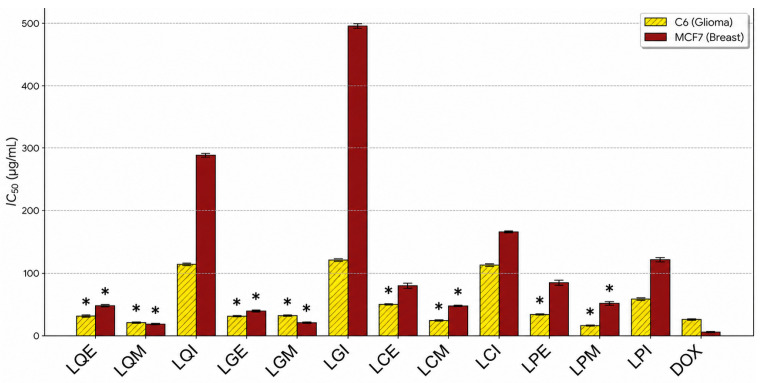
IC_50_ values of *Leonurus* extracts against C6 and MCF7 cell lines. Abbreviations: LQE, LGE, LCE, LPE: Ethanol extracts; LQM, LGM, LCM, LPM: Methanol extracts; LQI, LGI, LCI, LPI: Infusions of *L. quinquelobatus*, *L. glaucescens*, *L. cardiaca*, and *L. persicus*, respectively. DOX: Doxorubicin. Data represent mean ± SD (*n* = 3). * *p* < 0.05 vs. Doxorubicin.

To evaluate the antiproliferative potential of the extracts, the MTT method was applied against A549 (human lung carcinoma cell line), MCF7 (human breast adenocarcinoma cell line), and C6 (Rat glioma cell line) cell lines. Doxorubicin (DOX), used as a positive control. The results demonstrated that the antiproliferative effects were highly dependent on both the plant species and the extraction solvent (Figure 3). The IC_50_ values of the extracts are summarized in Table 5. Overall, the methanol extracts exhibited the lowest IC_50_ values, indicating a higher antiproliferative potency compared to the ethanol and infusion extracts. Among all tested samples, the methanol extract of *L. persicus* showed noteworthy activity against the C6 *glioma* cell line (IC_50_ 16.43 ± 0.17 µg/mL), which was comparable to or slightly more potent than the standard drug Doxorubicin (25.64 ± 1.11 µg/mL, *p* < 0.05) under the experimental conditions. Similarly, the methanol extracts of *L. quinquelobatus* and *L. glaucescens* demonstrated promising effects against MCF7 cells with IC_50_ values of 18.1 ± 0.45 µg/mL and 21.2 ± 0.93 µg/mL, respectively. Interestingly, most extracts showed limited efficacy (mostly >600 or >1000 µg/mL) against the A549 lung carcinoma line, suggesting a differential growth inhibitory profile. The MCF7 and C6 cells displayed higher sensitivity to the extracts compared to the A549 line. This differential response among different cancer phenotypes provides a preliminary basis for further investigation into the cell-specific mechanisms of *Leonurus* metabolites.

## 3. Discussion

HPLC-MS/MS analysis revealed that verbascoside, genkwanin, and caffeoylquinic acids (CQAs) are the predominant compounds in the studied extracts (Figure 1). Verbascoside, a prominent phenylethanoid glycoside, is well-documented for its multifaceted pharmacological profile, including significant anti-inflammatory, cardioprotective, and antioxidant properties, as well as its potential in tumor chemoprevention [17,18,19,20,21,22,23]. Genkwanin, a flavonoid recognized as a chemotaxonomic marker for the *Leonurus* genus, may correlate with the observed biological activities in these species [24]. This is particularly significant as our study provides the first detailed phytochemical mapping of these markers in *Leonurus* taxa native to Türkiye, highlighting regional chemical variations. Furthermore, CQAs, which are considered beneficial for human health primarily due to their antioxidant capacity, were consistently detected across the species [25]. It was observed that phenylethanoid glycosides (e.g., verbascoside) consistently exhibited higher signal intensities in methanol and ethanol extracts compared to infusions. On the other hand, the presence of these CQAs across all species may correlate with their consistent biological activity, while the variation in flavonoid and phenylethanoid levels may account for the differences in potency observed among the species. These findings imply that although the main components are widely present, their concentrations vary depending on the solvent.

The TPC analysis indicated prominent phenolic levels across all four *Leonurus* species. Based on our HPLC-MS/MS results, it can be suggested that phenylethanoid glycosides, specifically verbascoside, are the primary contributors to the total phenolic content due to their high relative abundance and multiple hydroxyl groups. Additionally, the presence of caffeoylquinic acids across all solvent systems further enhances the phenolic profile. Notably, in *L. persicus*, the presence of rosmarinic acid, which was not detected in the other three species, likely provides an additional contribution to its specific phenolic yield and antioxidant potential. This is supported by their high relative peak intensities observed in the chromatograms and the presence of multiple phenolic hydroxyl groups in their chemical structures, which typically yield strong responses in the Folin–Ciocalteu assay. Our findings align with previous phytochemical reports on *L. cardiaca*, which typically highlight an abundance of phenolic acids (e.g., chlorogenic and caffeic acids), flavonoids (rutin, quercetin), and phenylethanoid glycosides [11,26,27]. While earlier studies on the aerial parts of *L. glaucescens* reported the isolation of leonosides A and B along with lavandulifolioside [28], our analysis revealed a notable variation: lavandulifolioside was identified in *L. quinquelobatus*, *L. glaucescens*, and *L. cardiaca*, but was conspicuously absent in *L. persicus*. These qualitative differences in phenylethanoid distribution underscore the chemical diversity within Turkish *Leonurus* species and are consistent with the known complexity of the genus’s secondary metabolite profile.

Correlating these phytochemical profiles with pharmacological results provides a molecular basis for the reported activities. The differential sensitivity against MCF7 and C6 cancer cell lines (comparable to doxorubicin) may be associated with the presence of f genkwanin and verbascoside. Previous studies indicate that these compounds interfere with cellular proliferation pathways, which aligns with our findings of low toxicity toward A549 lung carcinoma cells [13]. Specifically, the prominent presence of genkwanin in the methanolic extract of *L. persicus* appears to be one of the key factors contributing to its anticandidal and cytotoxic effects. This flavonoid is recognized for its diverse repertoire, encompassing potent antibacterial [29,30], free radical scavenging [31], and chemopreventive properties [32].

The heatmap analysis (Figure 2) illustrates the correlation between total phenolic content (TPC) and antioxidant potency. The finding that all studied Turkish species meet the 0.2% flavonoid threshold of the European Pharmacopoeia is a noteworthy result, as it supports the quality and potential standardization of these local populations for pharmaceutical applications. Consequently, *L. cardiaca* stands out as a promising candidate for the development of standardized herbal products. Additionally, the antioxidant activity observed in infusions (aqueous extracts) provides a preliminary scientific basis for the traditional consumption of these plants as herbal teas in the region.

While the antibacterial activities observed were modest compared to isolated constituents like ursolic acid [33], the alcoholic extracts of *L. persicus* demonstrated a remarkably robust anticandidal effect. This activity is likely associated with the synergistic effect of genkwanin and verbascoside, alongside other minor constituents typical of the genus. Furthermore, *L. quinquelobatus* showed notable inhibition against Gram-negative *P. aeruginosa* in its methanolic form, suggesting that specific polar metabolites may contribute to overcoming the intrinsic resistance mechanisms of Gram-negative pathogens.

The cytotoxic results (Figure 3) are consistent with literature reporting that *L. sibiricus* extracts rich in phenolic compounds exhibit dose-dependent cytotoxicity against various cancer lines while sparing A549 cells [13,34]. Our data demonstrate a differential sensitivity among the tested cell lines, where Turkish *Leonurus* species exhibit suppressive effects on the viability of C6 glioma and MCF7 breast cancer cells compared to the lung epithelial-like A549 line. The cytotoxic activity of *L. persicus* methanol extract against C6 cells yielded an IC_50_ value of 16.43 ± 0.17 µg/mL. According to the criteria established by the U.S. National Cancer Institute (NCI), crude extracts with an IC_50_ < 20 µg/mL following 48 to 72 h of incubation are categorized as having notable cytotoxic activity [35]. Furthermore, extracts with IC_50_ values ranging from 21 to 200 µg/mL are classified as mildly cytotoxic, while those between 201 and 500 µg/mL are considered weakly cytotoxic [36]. Based on this standardized classification, our results indicate that while the alcoholic extracts of *L. persicus* exhibit a more pronounced effect against certain lines, the aqueous extracts (infusions) generally fall within the mildly cytotoxic range. While the IC_50_ values indicate notable growth inhibitory effects for the crude extracts, particularly the *L. persicus* methanol extract, against C6 cells, these should be interpreted as preliminary biological signals rather than direct drug-development leads. Phenolic compounds are known to modulate crucial cellular processes, including growth, differentiation, and apoptosis [37,38]. Among the identified compounds, genkwanin emerges as a noteworthy candidate for further investigation into its role in suppressing tumor growth [39,40,41]. However, it is essential to acknowledge that other characteristic metabolites, such as guanidino compounds (e.g., leonurine) which were not the primary focus of our current HPLC-MS/MS method, may also contribute to the overall bioactivity of these Turkish taxa. In conclusion, this study reveals for the first time the comparative cytotoxic effects of four *Leonurus* species native to Türkiye against different cell lines, establishing a potential connection between their regional phytochemical diversity and their health-promoting bioactivities. By documenting these local chemotypes, this study provides an essential foundation for the quality control and pharmacological validation of the Turkish flora. Furthermore, these results offer a useful foundation for more mechanistic research into the species-specific anti-cancer potential, even though they only constitute a preliminary screening of the populations under study.

## 4. Materials and Methods

### 4.1. Plant Materials and Extraction Procedures

The aerial parts of four *Leonurus* species were collected from their natural habitats in the Bursa, Bolu, and Erzurum provinces of Türkiye. The botanical authentication of the species was performed by Prof. Dr. Yavuz Bülent Köse and Dr. Nagehan Saltan. Voucher specimens have been deposited at the Anadolu University Faculty of Pharmacy Herbarium (ESSE). The collection localities and herbarium numbers are: *L. cardiaca*: Bursa, 40°02′21″ N, 29°23′57″ E (ESSE 15775), *L. quinquelobatus*: Bolu, 40°35′32″ N, 31°00′42″ E (ESSE 15780), *L. glaucescens*: Erzurum, 39°44′35″ N, 41°23′16″ E (ESSE 15783), *L. persicus*: Erzurum, 39°42′01″ N, 41°26′55″ E (ESSE 15782).

To compare the solvent efficiency and prepare the extracts for both phytochemical characterization and biological activity assays, two separate portions of the powdered material were extracted independently with 70% methanol (Sigma-Aldrich, St. Louis, MO, USA) and 70% ethanol (Sigma-Aldrich, St. Louis, MO, USA), respectively. Maceration was performed at room temperature for a total of 24 h in three repetitive cycles (8 h each) to ensure maximum metabolite recovery. The resulting filtrates were concentrated to dryness under vacuum using a rotary evaporator at a temperature not exceeding 40 °C. Residual water in the ethanol extracts was further eliminated through lyophilization. The third portion was used to prepare a 5% (*w*/*v*) infusion by adding 5 g of the plant material to 100 mL of distilled water preheated to 80 °C. Following a cooling and filtration period, the aqueous extract was frozen and subsequently dried using a lyophilizer. All obtained crude extracts were stored in amber glass vials at −18 °C in a dark environment until their use in both HPLC-MS/MS analysis and biological activity assays.

The extraction efficiency for each species was determined gravimetrically and expressed as a percentage yield (*w*/*w*) relative to the initial dried plant material. The yield was calculated using the following equation:

Yield (%)=(A÷A1)×100

A denotes the final mass of the dried extract obtained after solvent evaporation. A1 represents the initial mass of the pulverized plant material used in the extraction process.

### 4.2. Analysis of the Extracts by High-Performance Liquid Chromatography Systems (HPLC)

The analysis was conducted using a Shimadzu 20A HPLC system (Shimadzu, Tokyo, Japan) coupled to an Applied Biosystems 3200 Q-Trap MS/MS detector (3200 Q TRAP. Mundelein, IL, USA). Chromatographic separation was achieved on a 150 × 4.6 mm, 3 µm ODS column at a column temperature of 40 °C, and detection was performed with a PDA detector. The ionization mode for the MS/MS detector was set to negative Electro Spray Ionization (ESI). The mobile phase consisted of solvent (A) methanol/water/formic acid 10:89:1, (*v*/*v*/*v*) and solvent (B) methanol/water/formic acid (89:10:1, *v*/*v*/*v*). A gradient was applied, increasing the concentration of solvent B from 10% to 100% over 40 min. The flow rate was maintained at 0.5 mL/min.

The qualitative identification of the secondary metabolites was performed by a systematic interpretation of the resulting chromatograms and mass spectra. The identification process was achieved by correlating high-resolution mass data with established literature reports. Characterization was performed through the analysis of diagnostic fragmentation ions and neutral losses. For each identified constituent, the retention time (Rt), precursor ion [M-H]^-^, and diagnostic MS^2^ fragmentation patterns, such as the neutral losses of caffeoyl, glucose, or rhamnose moieties, were analyzed to ensure robust structural characterization (Table 1). The structural assignments were cross-referenced with previously published phytochemical profiles of the *Leonurus* genus to validate the qualitative fingerprinting of the Turkish taxa.

### 4.3. Total Phenolic (TPC) and Flavonoid (TFC) Content Assay

The total phenolic content (TPC) of the *Leonurus* extracts was determined using the Folin–Ciocalteu reagent (FCR) (Merck, Darmstadt, Germany) according to the method described by Singleton et al. [42]. This assay provides a measure of the total reducing capacity of the samples. For the quantification, a calibration curve was constructed using gallic acid as the standard at concentrations ranging from 0.03 to 0.5 mg/mL. The reaction mixtures were incubated under standardized conditions, and the absorbance was measured at 760 nm using a UV-Vis spectrophotometer (Shimadzu, Kyoto, Japan). TPC results were calculated from the linear regression equation derived from the standard curve and expressed as milligrams of gallic acid equivalents per gram of dry extract (mg GAE/g extract). All measurements were performed in triplicate to ensure reproducibility.

The total flavonoid content (TFC) was determined following the official spectrophotometric protocols established by the European Pharmacopoeia (Ph. Eur. 7.0) [7]. To ensure the reliability of the compliance test and to account for experimental variability, all measurements were performed in triplicate. For each *Leonurus* species, stock, test, and control solutions were prepared in accordance with the specific pharmacopoeial monographs. After an incubation period of 30 min at room temperature, the absorbance of the reaction mixture was measured at 425 nm. Absorbance values were recorded at the specified wavelength, and the results were expressed as a percentage of flavonoids calculated as hyperoside or equivalent markers as defined by the Ph. Eur. standards. The percentage of total flavonoids, calculated as hyperoside equivalents, was determined using the following equation:

$$TFC(\%)=\frac{A\times 1.25}{m}$$

A: Absorbance at 425 nm;
m: Mass of the substance being analysed (in grams).

### 4.4. DPPH^•^ and ABTS^•+^ Scavenging Capacity Determination

The DPPH^•^ radical scavenging activity was determined according to the previously described method [43], using gallic acid as a positive control. After the incubation period, the absorbance of the reaction mixtures was measured at 517 nm. The percentage of radical scavenging activity was calculated using the following equation:

$$Scavenging\;effect(\%)\left[\frac{A_{control}-A_{sample}}{A_{control}}\right]\times 100$$

where A_control_ is the absorbance of the control (containing all reagents except the test compound), and A_sample_ is the absorbance of the test sample. The EC_50_ (Half-maximal effective concentration) values were derived from non-linear regression analysis using SigmaPlot software (version 13.0, SPSS Inc., Chicago, IL, USA). All experiments were performed in triplicate, and the data are presented as mean values.

The ABTS^•+^ radical cation scavenging activity was determined according to the method described by Re et al. [44]. The ABTS^•+^ solution was prepared and diluted to an absorbance of 0.70 ± 0.02 at 734 nm. Trolox was employed as the standard reference, with a calibration curve generated using concentrations ranging from 0.25 to 3 mM. The reaction mixtures were incubated for 30 min, after which the absorbance was measured at 734 nm. The scavenging capacity of the samples (tested at a concentration of 2.5 mg/mL) was calculated using the linear regression equation derived from the Trolox standard curve. Results are expressed as Trolox Equivalent Antioxidant Capacity (TEAC) values. All measurements were performed in triplicate to ensure reproducibility, and the data are presented as means ± standard deviation (SD).

### 4.5. Antimicrobial Activity

Six strains of invasive fungal infections caused by *Candida* and six strains of other pathogenic bacteria were employed as test microorganisms (Table 3). Antibacterial susceptibility was determined using the broth microdilution method according to the Clinical and Laboratory Standards Institute (CLSI) M7-A7 guidelines [45]. Anticandidal activities were assessed following the adapted CLSI M27-A2 standard protocol [46]. Stock solutions of extracts and antimicrobial agents (Amphotericin-B, Ketoconazole, Chloramphenicol, Ampicillin) were dissolved in DMSO. The M27-A2 susceptibility testing of yeasts procedure employed *C. parapsilosis* ATCC 22019 and *C. krusei* ATCC 6258 as quality control strains. The extracts were tested at concentrations ranging from 8 mg/mL to 0.001 mg/mL. The Minimum Inhibitory Concentration (MIC) was defined as the lowest concentration of the extract that prevented visible microbial growth compared to the control. The growth controls were microplates without antimicrobial standards. To validate sterility and the effects of the solvents, negative controls were used. DMSO-containing wells were used as negative controls to ensure sterility and evaluate solvent effects. All assays were performed in duplicate.

### 4.6. Antiproliferative Activity

The cytotoxic potential of the extracts was evaluated using the MTT (3-(4,5-dimethylthiazol-2-yl)-2,5-diphenyltetrazolium bromide) assay, as previously described [47], with Doxorubicin employed as the reference drug. Human lung carcinoma (A549), rat glioma (C6), and human breast adenocarcinoma (MCF-7) cell lines were obtained from the American Type Culture Collection (ATCC, Manassas, VA, USA) and cultured in RPMI-1640 medium supplemented with 10% fetal bovine serum (FBS) and 1% penicillin/streptomycin (100 U/mL and 100 µg/mL, respectively). Cells were collected and quantified using an automated cell counter. After cell adhesion, cells were seeded in 96-well plates at a density of 1 × 10^4^ per well, and treated with varying concentrations of the extracts (7.81–1000 µg/mL). Following 24 h of incubation, MTT solution (5 mg/mL, prepared in PBS) was added to each well. After the formation of formazan crystals, the absorbance was measured at 540 nm using a Synergy H1 microplate reader (BioTek Instruments, Winooski, VT, USA). To ensure the reliability of the results, cell-free control wells (extracts + MTT without cells) were utilized to monitor potential chemical interference; no significant background signal was detected. All samples were dissolved in DMSO, with 1% DMSO used as the negative control. The IC_50_ values were determined from dose–response curves (percentage of inhibition vs. log concentration) generated via non-linear regression analysis using Microsoft Excel 2013. The percentage of inhibition was determined using the formula previously described in the DPPH assay section.

### 4.7. Statistical Analysis and Data Visualization

All experimental measurements were conducted in triplicate, and data are presented as mean ± standard deviation (SD). The normality of the data was confirmed using the Shapiro–Wilk test prior to ANOVA analysis. Statistical significance was determined by one-way analysis of variance (ANOVA) followed by Tukey’s post hoc test using IBM SPSS Statistics (version 24.0; IBM Corp., Armonk, NY, USA). Pearson correlation coefficients were calculated to determine the relationship between TPC and antioxidant assays (DPPH and TEAC). The heatmap and comparative bar charts were generated using Python (version 3.10) with Seaborn (v0.12) and Matplotlib (v3.5) libraries for high-resolution data visualization. For the heatmaps, data were normalized using Z-score transformation to ensure comparability across different biological assays.

## 5. Conclusions

This study provides a comprehensive and pioneering characterization of four *Leonurus* species (*L. cardiaca*, *L. quinquelobatus*, *L. glaucescens*, and *L. persicus*) naturally occurring in Türkiye, marking the first detailed comparative report on their phytochemical profiles and biological capacities. Our findings reveal that the therapeutic potential of these species is intrinsically linked to their secondary metabolite composition, specifically the presence of verbascoside, genkwanin, and caffeoylquinic acids identified via HPLC-MS/MS.

The observed correlation between the total phenolic content and the antioxidant/antimicrobial activities, particularly in *L. cardiaca*, suggests the role of these polyphenols as key bioactive drivers. Furthermore, the extracts exhibited varying levels of susceptibility against MCF7 and C6 cell lines, while maintaining lower cytotoxicity toward A549 cells under the tested conditions. These preliminary results position these extracts as candidates for further research to explore their differential growth inhibitory effects compared to standard treatments.

In conclusion, this systematic investigation provides scientific evidence supporting the traditional medicinal use of *Leonurus* species and highlights their potential as standardized sources of bioactive compounds for the pharmaceutical and nutraceutical sectors. These results lay a robust foundation for future bioactivity-guided isolation studies to further elucidate the molecular mechanisms behind their differential biological properties.

## Figures and Tables

**Table 1:** Phytochemical screening of main bioactive compounds in Turkish *Leonurus* extracts via HPLC-MS/MS.

	Rt (min)	[M−H]^−^ (*m/z*)	Main MS^2^ Fragments (*m/z*)	Compound	*L. persicus*	*L. glaucescens*	*L. quinquelobatus*	*L. cardiaca*
1	12.5	352.7	191, 179, 173	Caffeoylquinic acid	E, M, I	E, I	E, M, I	E, M, I
2	12.8	352.7	191, 179, 135	5-O-Caffeoylquinic acid	E, M, I	E, I	–	–
3	15.7	178.8	135	Caffeic acid	E, M, I	E, I	E, M, I	E, M, I
4	16.1	754.4	623, 462, 305, 179, 161, 135	Lavandulifolioside	–	E, I	E, M, I	E, M, I
5	16.5	622.4	461, 161, 135	Verbascoside	E, M, I	E, M, I	E, M	E, M
6	18.0	358.7	197, 179, 161	Rosmarinic acid	E, M, I	–	–	–
7	19.9	608.4	300, 271, 179, 151	Quercetin rutinoside	E, M, I	E, M, I	E, M, I	E, M, I
8	20.2	462.6	300, 271, 255	Quercetin glucoside	–	E, M	E, M, I	E, M, I
9	32.2	282.7	268, 239, 211, 151, 117	Genkwanin	E, M, I	E	E, M	E, M

[E] Ethanol extract. [M] Methanol Extract. [I] Infusion (Aqueous extract). Note: Only major compounds with confirmed MS/MS data and library match scores >90% are listed.

**Table 2:** Extraction yield, total phenolic content (TPC), total flavonoid content (TFC), and antioxidant activities of *Leonurus* species extracts.

Samples	Yield%	TPC mg GAE/g Ext.	DPPH˙(EC_50_ mg/mL)	TEAC (mM/Trolox)
*L. quinquelobatus*				
EtOH	11	81.15 ± 0.02	0.218 ± 0.11	1.535 ± 0.14
MeOH	6.6	41.9 ± 0.01	0.552 ± 0.03	0.646 ± 0.04
Infusion	7.8	78.8 ± 0.04	0.166 ± 0.02	1.179 ± 0.04
*L. glaucescens*				
EtOH	10	143.4 ± 0.03	0.133 ± 0.01	2.53 ± 0.01
MeOH	10.3	107.8 ± 0.02	0.391 ± 0.10	0.778 ± 0.02
Infusion	13.6	74 ± 0.01	0.155 ± 0.01	1.04 ± 0.02
*L. cardiaca*				
EtOH	9.3	170.1 ± 0.02	0.117 ± 0.01	2.731 ± 0.01
MeOH	13.3	48.4 ± 0.02	0.256 ± 0.02	0.992 ± 0.05
Infusion	13.8	72 ± 0.02	0.200 ± 0.02	1.15 ± 0.04
*L. persicus*				
EtOH	10.6	148.1 ± 0.04	0.182 ± 0.04	2.251 ± 0.05
MeOH	10.5	70.2 ± 0.03	0.629 ± 0.03	0.633 ± 0.03
Infusion	15.6	78 ± 0.01	0.232 ± 0.03	1.207 ± 0.02
GA (gallic acid)			0.003 ± 0.001	2.926 ± 0.03
**TFC (%)**				
**Species**		**Absorbance**	**% Flavonoid**	
*L. quinquelobatus*		0.214	0.26 ± 0.05	
*L. glaucescens*		0.284	0.35 ± 0.03	
*L. cardiaca*		0.196	0.24 ± 0.02	
*L. persicus*		0.255	0.31 ± 0.03	

Values are means of triplicate determination (*n* = 3) ± standard deviation.

**Table 3:** Antibacterial activity of extracts (MIC, µg/mL).

	Ec	Saa	See	Ef	Se	Pa
*L. quinquelobatus*						
EtOH	2000	2000	2000	2000	2000	>8000
MeOH	2000	2000	2000	1000	1000	4000
Inf	2000	>8000	2000	2000	2000	>8000
*L. glaucescens*						
EtOH	2000	>8000	2000	2000	2000	>8000
MeOH	2000	1000	2000	1000	2000	>8000
Inf	2000	4000	2000	2000	2000	>8000
*L. cardiaca*						
EtOH	2000	4000	2000	2000	2000	>8000
MeOH	2000	2000	2000	1000	2000	>8000
Inf	2000	4000	2000	2000	2000	>8000
*L. persicus*						
EtOH	2000	2000	2000	2000	2000	>8000
MeOH	2000	2000	2000	2000	2000	>8000
Inf	2000	4000	2000	2000	2000	>8000
Ampicillin	4	2	2	4	4	128
Chloramphenicol	4	4	4	4	4	64

Ec: *E. coli* ATCC 8739, Saa: *S. aureus* subsp. *aureus* ATCC 6538, See: *S. enterica* subsp. *enterica* ATCC 14028, Ef: *E. faecalis* ATCC 29212, Se: *S. epidermidis* ATCC 12228, Pa: *P. aeruginosa* ATCC 7853, Inf: Infusion, MIC: Minimum inhibitor concentration.

**Table 4:** Anticandidal activity of extracts (MIC, µg/mL).

Extracts	Ca1	Cu	Ca2	Ct	Cp	Ck
*L. quinquelobatus*						
EtOH	1000	500	1000	1000	1000	500
MeOH	1000	500	1000	500	500	250
Inf	2000	1000	2000	1000	1000	500
*L. glaucescens*						
EtOH	1000	500	1000	1000	1000	250
MeOH	1000	500	500	1000	500	250
Inf	1000	1000	1000	2000	500	500
*L. cardiaca*						
EtOH	500	250	500	1000	500	250
MeOH	500	250	500	500	500	500
Inf	2000	1000	2000	2000	2000	2000
*L. persicus*						
EtOH	500	250	500	500	125	125
MeOH	250	125	500	250	500	125
Inf	1000	500	1000	2000	1000	500
Amphotericin-B	0.25	0.125	0.5	0.25	0.25	0.25
Ketoconazole	0.06	0.03	0.03	0.03	0.03	0.06

Ca1: *C. albicans* ATCC 10231, Cu: *C. utilis* NRRL Y-900, Ca2: *C. albicans* ATCC 90028, Ct: *C. tropicalis* ATCC 750, Cp: *C. parapsilosis* ATCC 22019, Ck: *C. krusei* ATCC 6258, Inf: Infusion, MIC: Minimum inhibitor concentration.

**Table 5:** Antiproliferative activity of *Leonurus* extracts against cancer cell lines (µg/mL).

Cell Lines			
Extracts/Standard	A549	C6	MCF7
*L. quinquelobatus*			
EtOH	>1000	31.33 ± 1.39	47.81 ± 2.08
MeOH	>1000	21.14 ± 1.15	18.19 ± 0.45
Inf	>1000	114.78 ± 1.39	288.61 ± 2.65
*L. glaucescens*			
EtOH	>1000	31.22 ± 0.60	39.26 ± 1.65
MeOH	>1000	32.38 ± 0.27	21.22 ± 0.93
Inf	>1000	121.76 ± 1.39	495.87 ± 3.62
*L. cardiaca*			
EtOH	869.56 ± 10.5	50.12 ± 1.09	80.65 ± 3.94
MeOH	673.67 ± 9.85	24.31 ± 1.03	47.59 ± 1.38
Inf	>1000	113.64 ± 1.39	165.91 ± 1.56
*L. persicus*			
EtOH	>1000	33.96 ± 0.39	85.65 ± 3.71
MeOH	601.86 ± 8.66	16.43 ± 0.17	51.79 ± 2.36
Inf	>1000	58.93 ± 1.39	122.27 ± 2.54
DOX	10.76 ± 0.513	25.64 ± 1.11	5.70 ± 0.22

DOX: Doxorubicin; Inf: Infusion. IC_50_ values are given as mean values ± SDs of three independent measures.

## Data Availability

The original contributions presented in this study are included in the article/Supplementary Materials. Further inquiries can be directed to the corresponding author.

## Supplementary Materials

The following supporting information can be downloaded at: https://www.mdpi.com/article/10.3390/molecules31101708/s1. Figure S1: Verbascoside. Figure S2: Lavandulifolioside. Figure S3: Genkwanin. Figure S4: 5-O-Caffeoylquinic acid. Figure S5: Caffeic acid. Figure S6: Rosmarinic acid. Figure S7: Quercetin rutinoside. Figure S8: Quercetin glucoside. Figure S9: HPLC-DAD (Diode Array Detector) chromatograms (recorded at 330 nm) of *L*. *cardiaca*. Figure S10: HPLC-DAD (Diode Array Detector) chromatograms (recorded at 330 nm) of *L. glaucescens.* Figure S11: HPLC-DAD (Diode Array Detector) chromatograms (recorded at 330 nm) of *L. quinquelobatus.* Figure S12: HPLC-DAD (Diode Array Detector) chromatograms (recorded at 330 nm) of *L. persicus*.

## Abbreviations

ABTS	2,2′-azino-bis (3-ethylbenzothiazoline-6-sulfonic acid)
ATCC	American Type Culture Collection
DPPH	2,2-diphenyl-1-picrylhydrazyl
CLSI	Clinical and Laboratory Standards Institute
DMSO	Dimethyl Sulfoxide
EtOH	Ethanol
TEAC	Trolox Equivalent Antioxidant Capacity
MeOH	Methanol
mM	Millimolar
Na_2_CO_3_	Sodium Carbonate
EC_50_	Half-maximal effective concentration
MIC	Minimum Inhibitory Concentration
TPC	Total Phenol Content
TFC	Total Flavonoid Content
