# Supplementary material for: Chemical Composition and Bioactivities of Turkish Leonurus Species (Lamiaceae) Extracts: Antioxidant, Antimicrobial, and Antiproliferative Potential
Source: Molecules. 2026 May 18;31(10):1708. doi: 10.3390/molecules31101708 (PMC13209506; doi:10.3390/molecules31101708)
Supplement: Supplementary file 1 [file molecules-31-01708-s001.zip › molecules-4262293-supplementary.pdf]

Chromatograms of *Leonurus* species

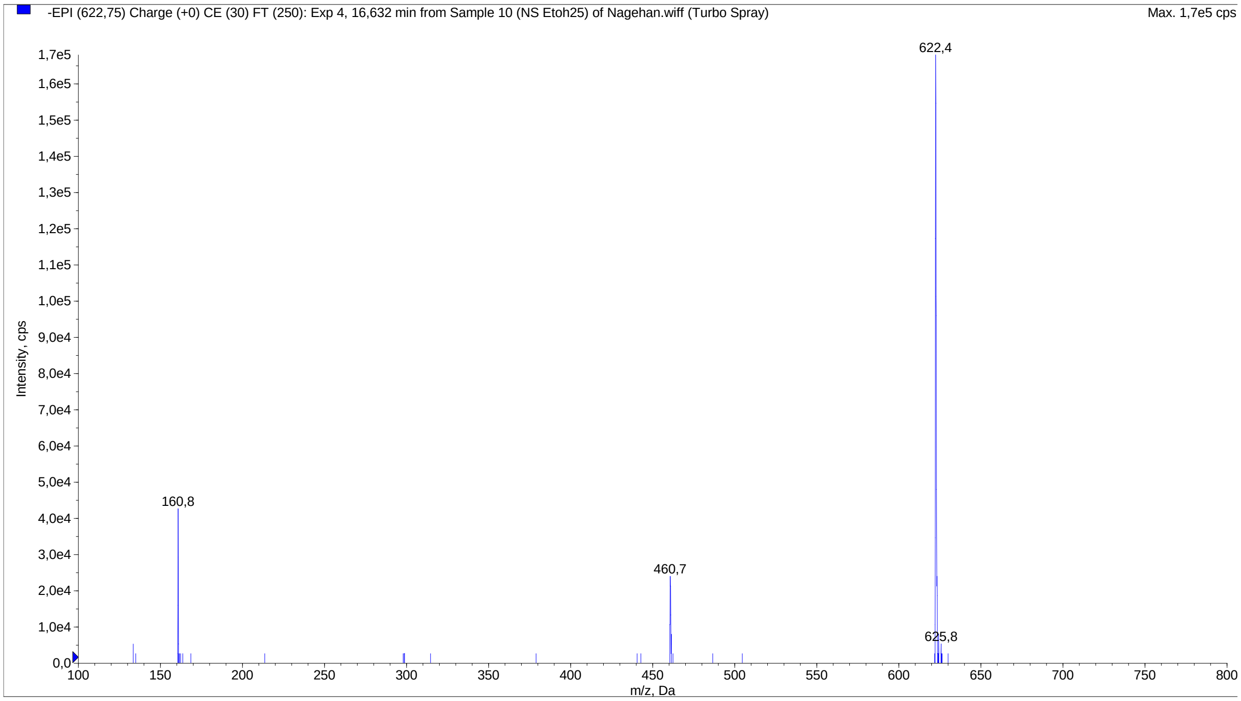

Figure S1. Verbascoside

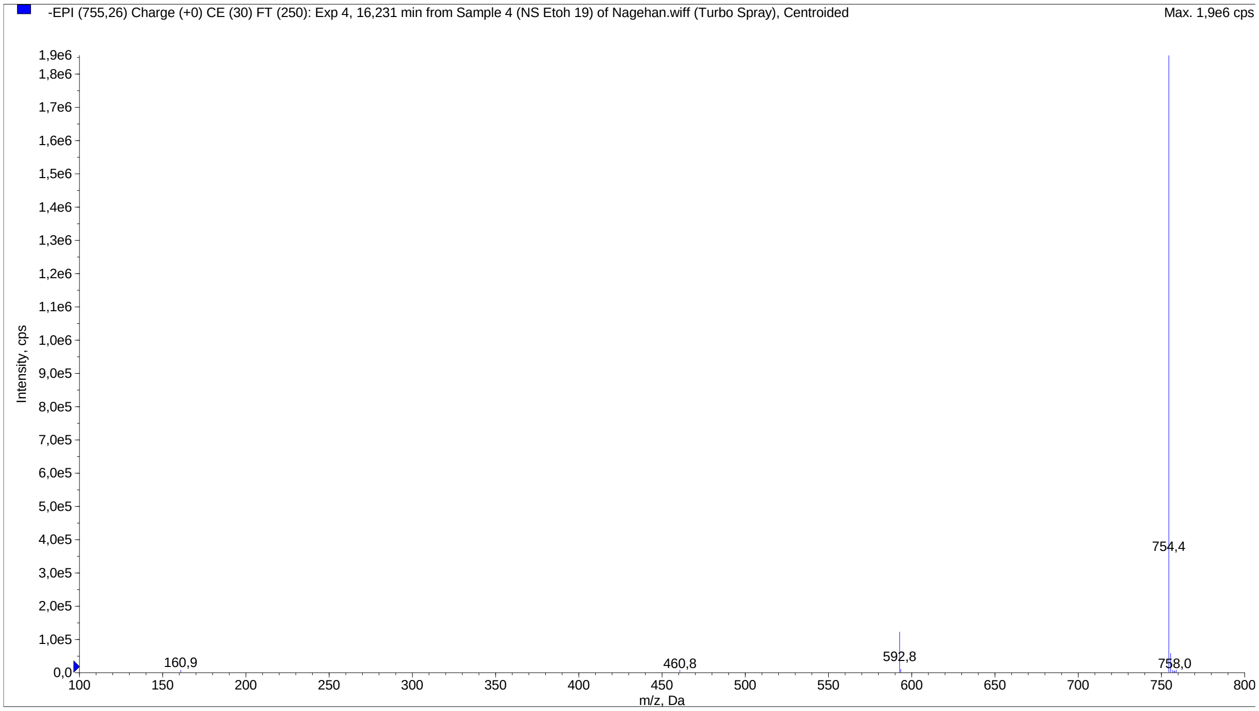

Figure S2. Lavandulifolioside

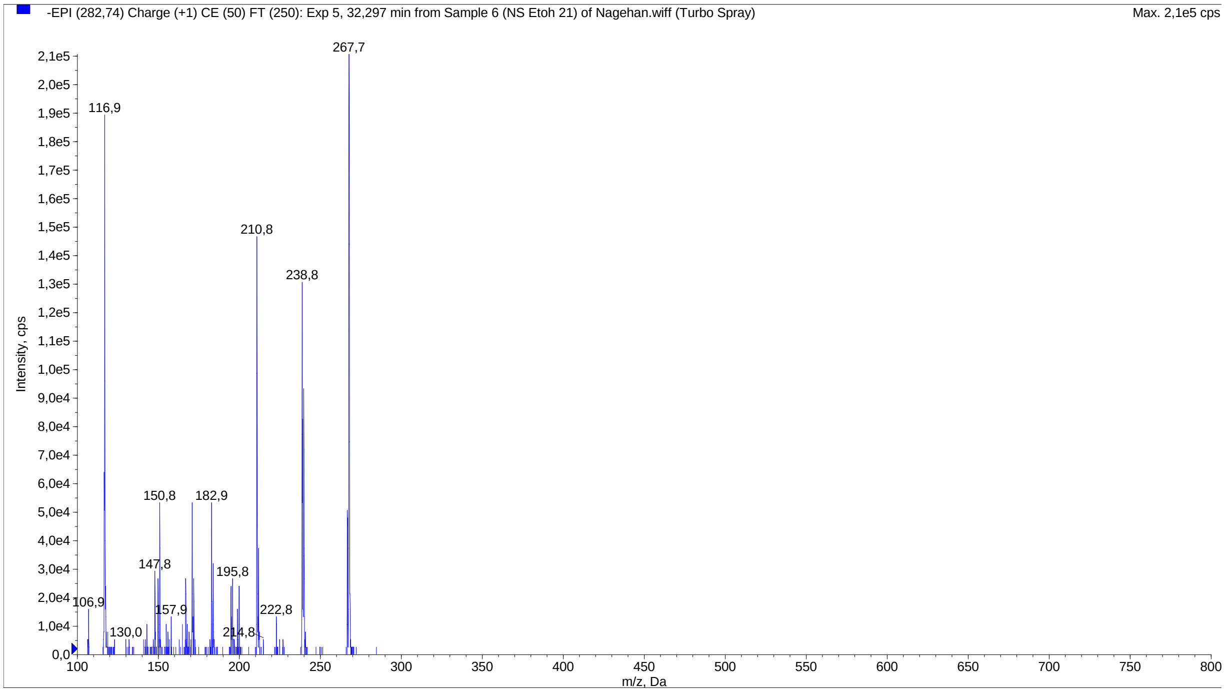

Figure S3. Genkwanin

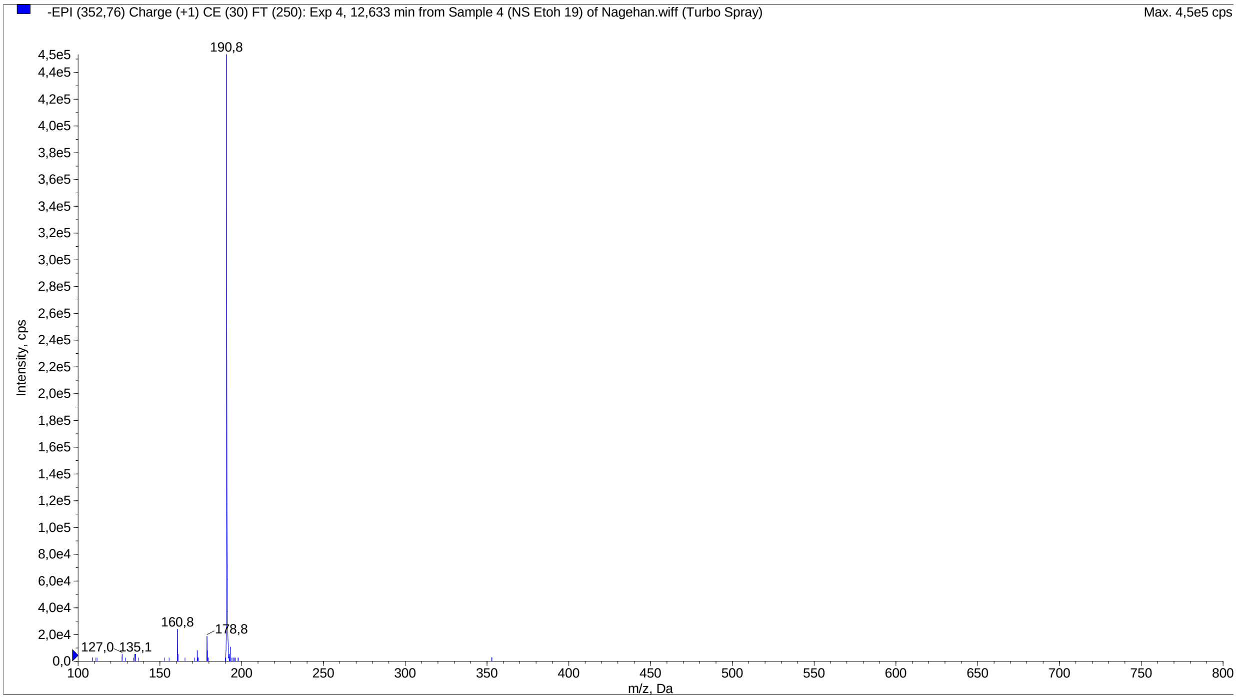

Figure S4. 5-O-Caffeoylquinic acid

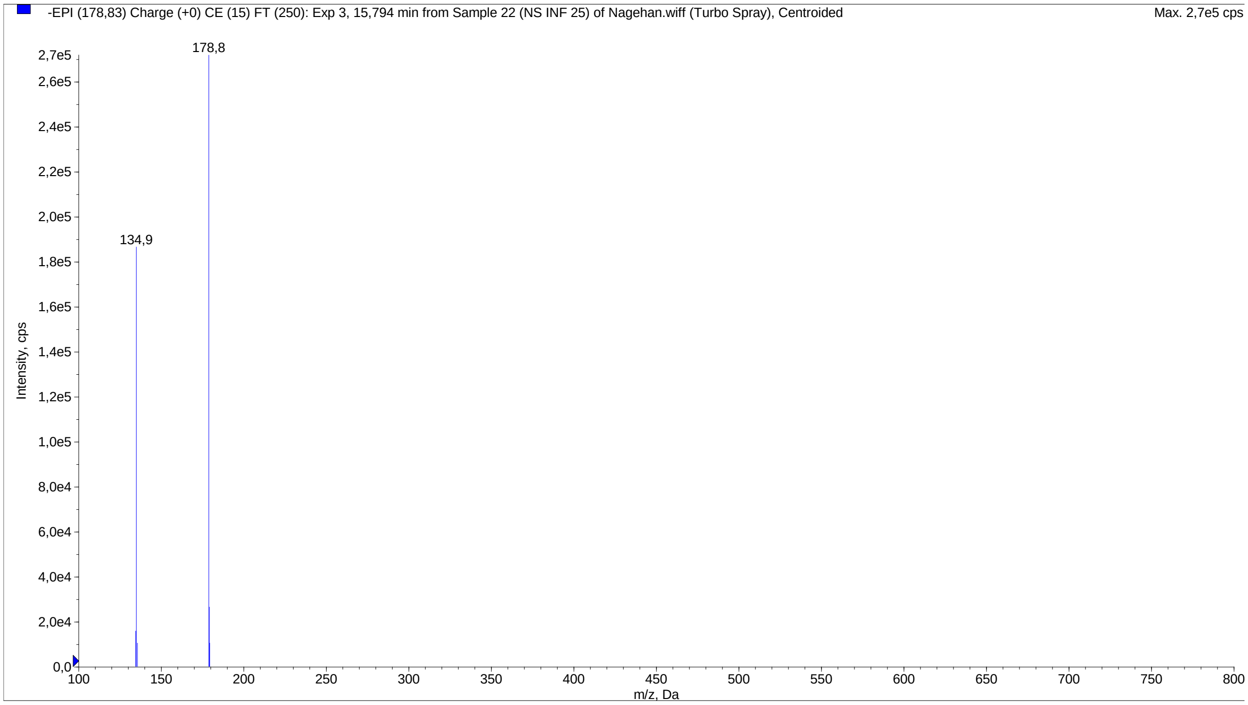

Figure 5. Caffeic acid

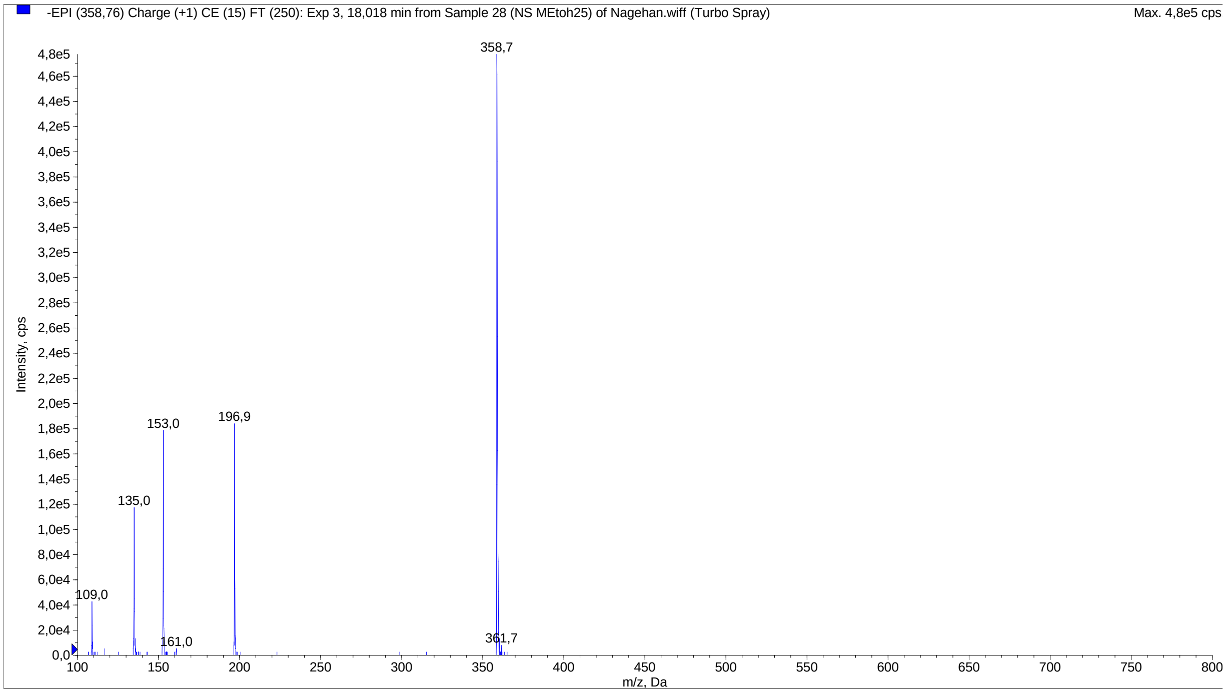

Figure S6. Rosmarinic acid

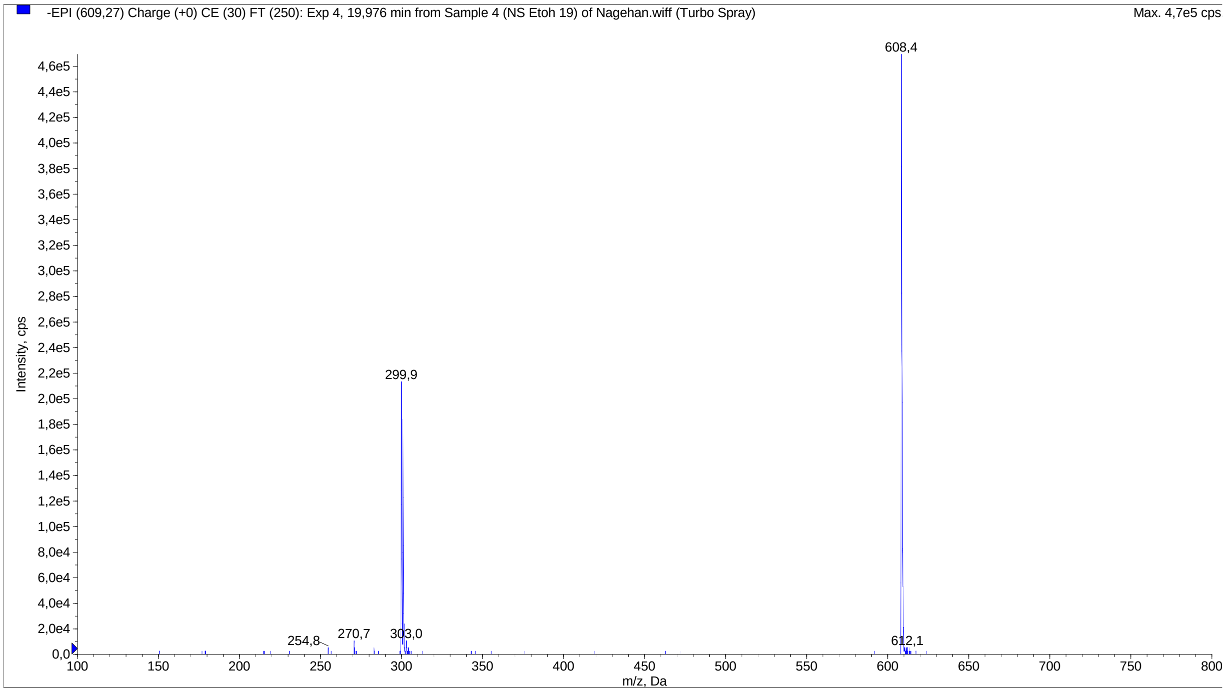

Figure S7. Quercetin rutinoside

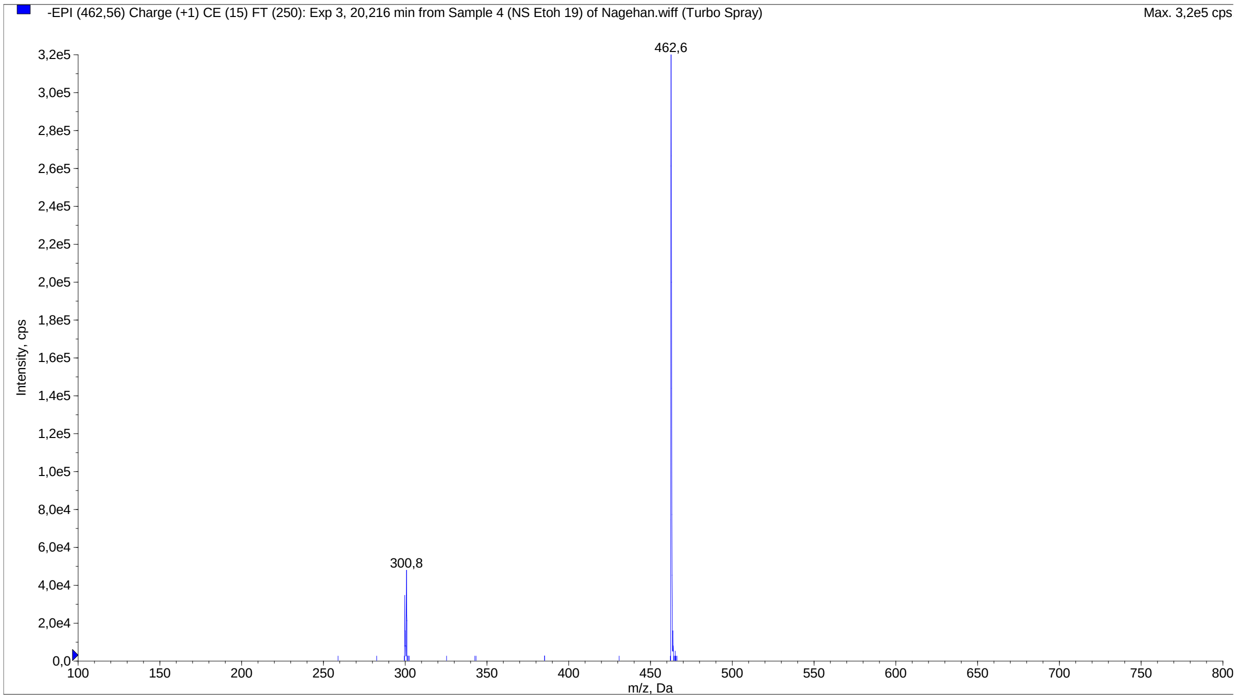

Figure S8. Quercetin glucoside

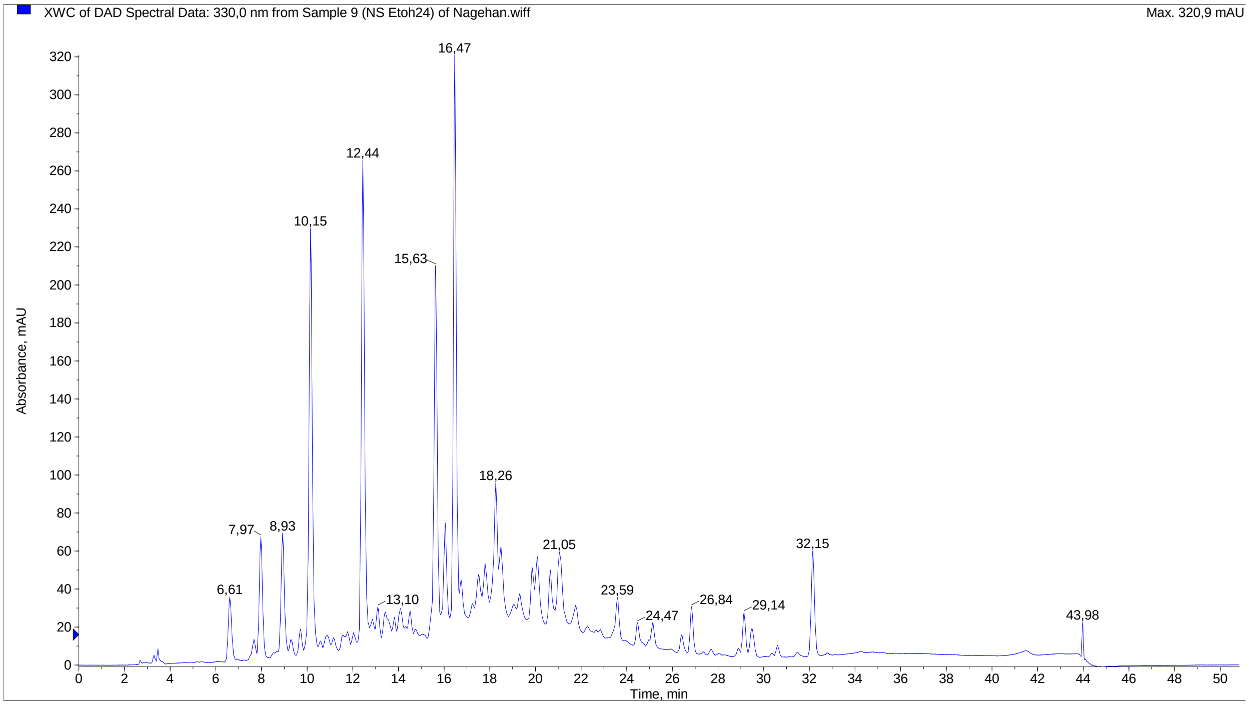

Figure S9. HPLC-DAD (Diode Array Detector) chromatograms (recorded at 330 nm) of *L. cardiaca*

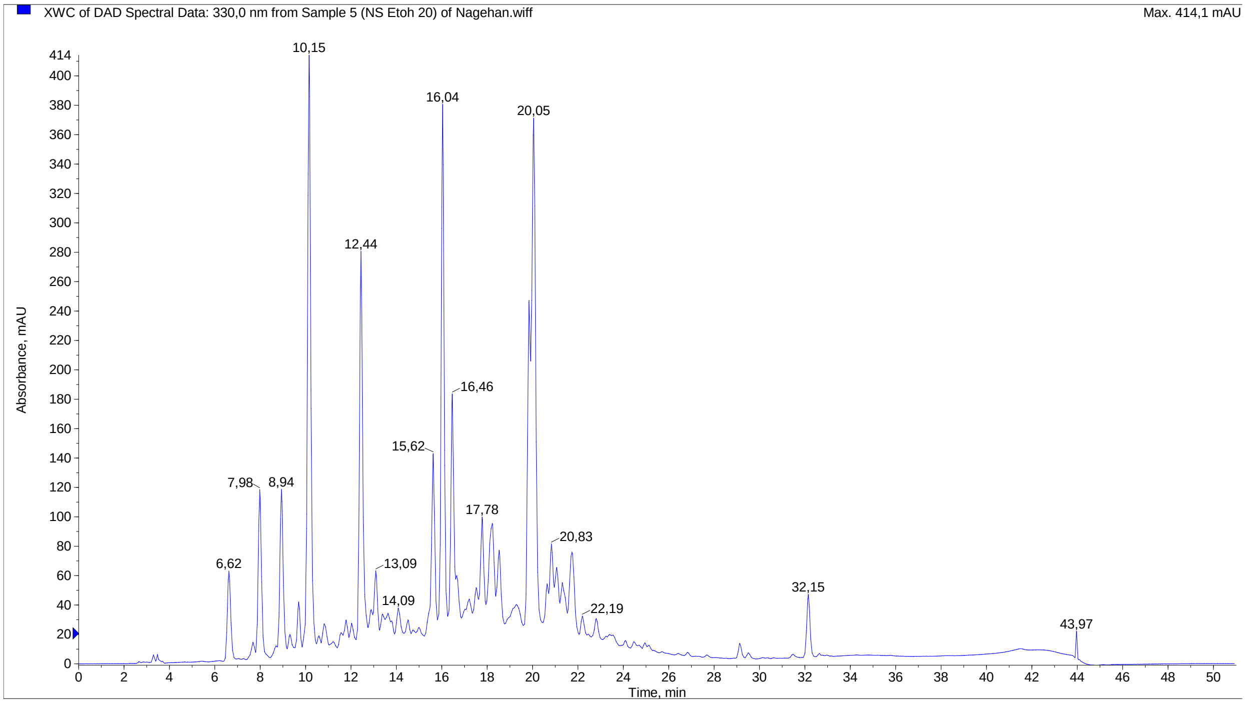

Figure S10. HPLC-DAD (Diode Array Detector) chromatograms (recorded at 330 nm) of *L. glaucescens*

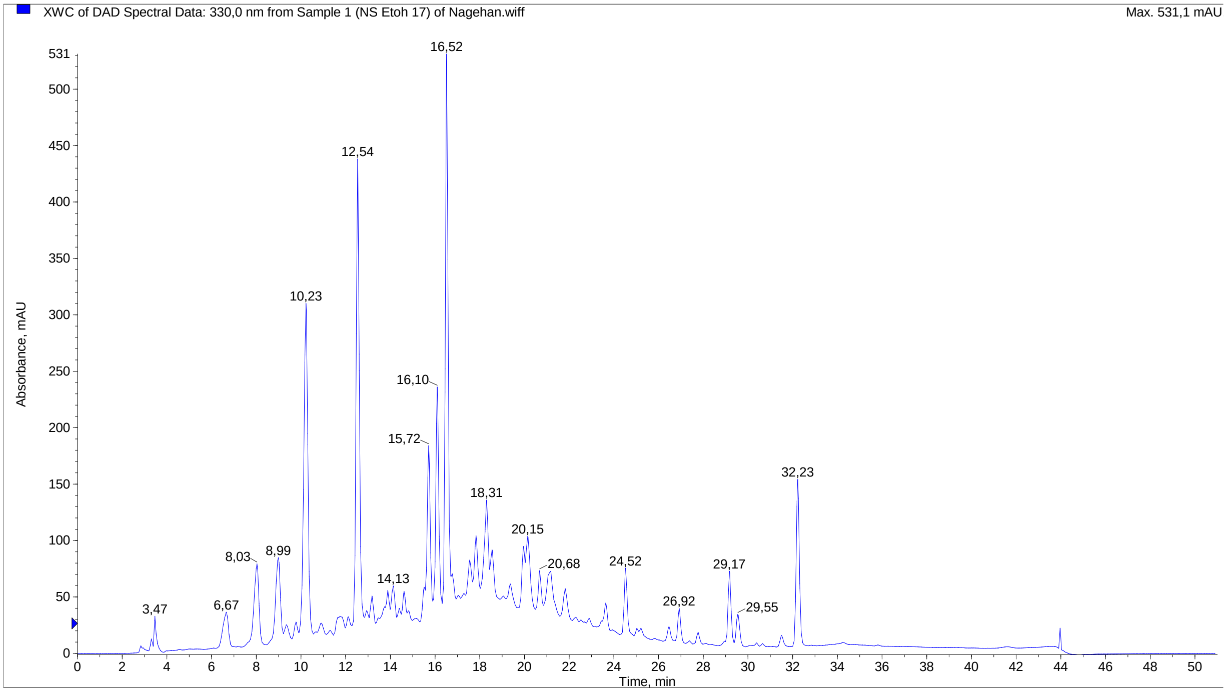

Figure S11. HPLC-DAD (Diode Array Detector) chromatograms (recorded at 330 nm) of *L. quinquelobatus*

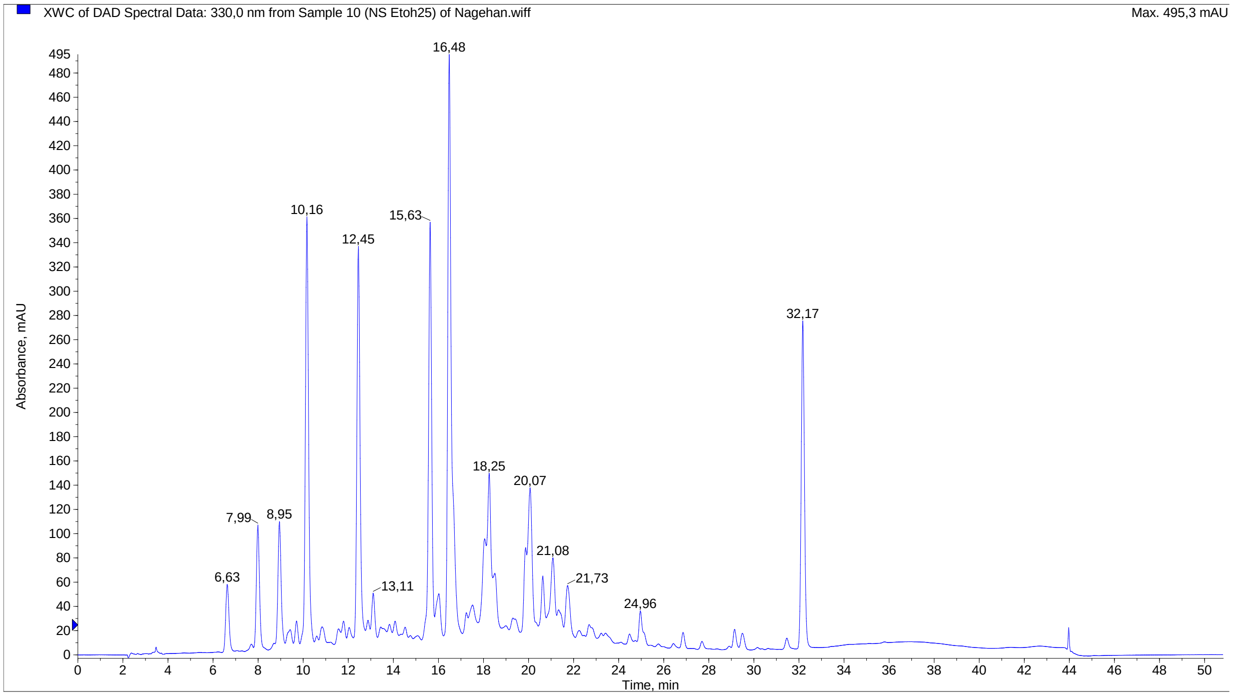

Figure S12. HPLC-DAD (Diode Array Detector) chromatograms (recorded at 330 nm) of *L. persicus*
